# Air-Bubble Induced Mixing: A Fluidic Mixer Chip

**DOI:** 10.3390/mi11020195

**Published:** 2020-02-14

**Authors:** Xiaoyu Jia, Bingchen Che, Guangyin Jing, Ce Zhang

**Affiliations:** 1 School of Physics, Northwest University, Xi’an 710069, China; 201720556@stumail.nwu.edu.cn; 2 State Key Laboratory of Cultivation Base for Photoelectric Technology and Functional Materials, Institute of Photonics and Photon-Technology, Northwest University, Xi’an 710069, China; bingchenc03@gmail.com

**Keywords:** fluidic mixer, air bubble, 3D printing

## Abstract

In this study, we report the design and fabrication of a novel fluidic mixer. As proof-of-concept, the laminar flow in the main channel is firstly filled with small air-bubbles, which act as active stirrers inducing chaotic convective turbulent flow, and thus enhance the solutes mixing even at a low input flow rate. To further increase mixing efficiency, a design of neck constriction is included, which changes the relative positions of the inclusion bubbles significantly. The redistribution of liquid volume among bubbles then causes complex flow profile, which further enhances mixing. This work demonstrates a unique approach of utilizing air bubbles to facilitate mixing in bulk solution, which can find the potential applications in microfluidics, fast medical analysis, and biochemical synthesis.

## 1. Introduction

Mixing of the solutes in fluid with an efficient way is essential in chemical, biological, medical, and material industries [1,2,3,4,5,6]. A fluidic mixer is not only a crucial component for lab-on-a-chip studies, but also has great practical applications [7,8]. The uniform laminar flow is the characteristic feature of the fluidic channels, which use basic diffusion process plus the sharp wedge for the passive slow mixing [9]. For practical use, it is highly desirable to mix the solute across the channel section before the solute is transported forward. To accomplish fully chaotic mixing, there emerged many fluidic mixers over the years, which can be cataloged into two types: the passive and the active mixer. The passive mixer utilizes geometric obstacles to create the flow field perturbation [10,11,12,13,14], such as intersection channels [15,16], convergent–divergent channels [17], three-dimensional architectures by multiple fabrications of the channels [18,19], and embedded, barrier-based obstacles [20]. Curved wall of the channel is better to disturb the flow than the flat straight one. Periodic wavy poly-dimethylsiloxane (PDMS) channels was designed to enhance the mixing process due to the vortex generation located at the higher curved part [21]. The active fluidic mixer induces external energy sources, which can be mechanical pulsation or electrokinetic forces [22,23,24,25,26]. Even though active fluidic mixer is favorable due to many adjustable parameters (e.g., perturbation frequency, phase, and amplitude), only a few studies have been reported due to the complexity of control schemes and difficulties in fabrications. The droplet mixer generates chaotic advection by pressure-driving a flow cavity (droplet) through fluidic channels of various geometries [27,28]. Relative motion of the surrounding walls induces convection flows that mix solutions. As a passive mixer, the mixing mostly depends on the geometry of fluidic channels, which determines whether the solute can pass across the streamlines. Therefore, there may still be islands within the flow cavity, where the solute is not touchable. A combination of channel geometry and the inclusion of a slip boundary into the flow, i.e., emulsions or bubbly flow, is believed to be demanding for mixing in microfluidic channels. We herein present a simple design, using air-bubbles as the stirrer. We propose that the presence of bubbles within the otherwise laminar flow can stimulate a fascinating variety of motion patterns on one hand, and the instabilities to transfer fluid momentum with the uniformization process on the other hand.

## 2. Fabrication and Experiments

### 2.1. Chip Fabrication

**Figure 1:**
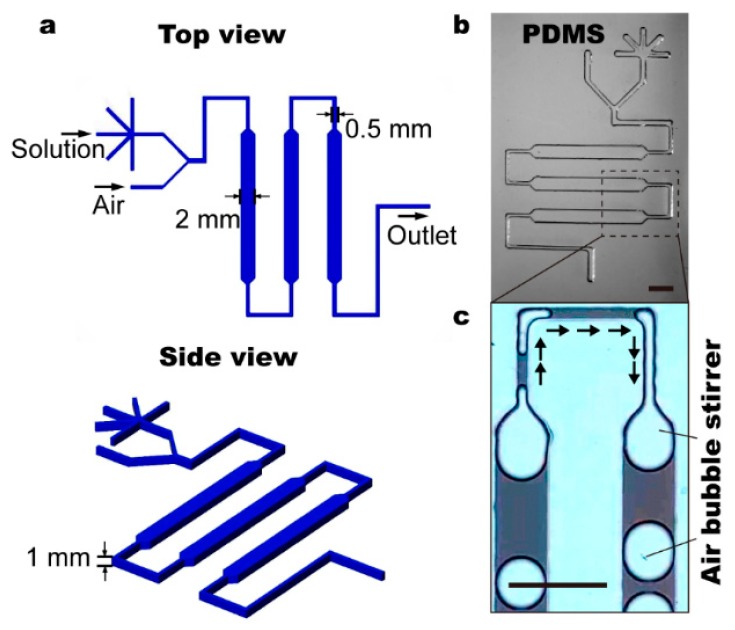
Schematic drawing (**a**) and optical image (**b**) of the fluidic mixer. (**c**) Air bubbles generated on-chip and transport within fluidic channels. Scale bars denote 4 mm in all figures.

Chip Fabrication was performed using the standard soft lithography approach in combination with 3D printing (Figure S1) [29]. The poly-dimethylsiloxane (PDMS) device can consist of one or multiple layers, depending on the chip functionality. The 3D-printer (CR-3040, CREALITY, Shenzhen, China), which can produce structures with a minimum width of 0.4 mm and a minimum height of 0.1 mm, was used to create negative templates for each layer. The 3D-printed template surface was smoothed with alcohol before PDMS casting. To fabricate fluidic chips with only one layer, the 3D-printed mold was cast with a PDMS mixture with a curing agent at 5:1, and cured by incubating at 45 °C for 1 h to 2 h depending on the thickness. Subsequently, the resulting PDMS duplicated get holes punched where the gas and solution inputs were connected (see Figure 1). The fluidic device was sealed using glass slides, which were spin-coated with a 20:1 PDMS mixer at 1200 rpm and cured at 80 °C for 1 h. Following standard PDMS-PDMS bonding protocol (i.e., off-ratio bonding), the PDMS devices (5:1) and PDMS coated glass slides (20:1) were brought in contact and incubated at 80 °C for 24 h before use. For multilayer fluidic devices (e.g., the one shown in Figure S2), the same protocol applied with alternative 20:1 and 5:1 mixing ratios starting from the top flow layer.

### 2.2. Chip Control and Operation

To set up a chip, we connected the PDMS chips to tubes filled with DI-water via TYGON tubing (Milan, Satigny, Switzerland). In order to degas the chip, the outlet was firstly sealed and air was fully pressed out through the porous PDMS material, while fluid remained in the chip. The process continued till the flow layer was fully filled by DI water. During experiments, 4 inlets were connected to food dyes of different colors, and one inlet was connected to a pressurized gas pipeline.

### 2.3. Data Analysis

To quantify the color transition during mixing, we split the optical images into red, green and blue mono-color channels. We picked the red channel, in which the intensity difference reflects the color transitions during mixing. Intensity variances at different positions along the fluidic channel were collected, and plotted against the distance away from the inlet, as illustrated in Figure S3.

## 3. Results and Discussion

### 3.1. The Theory

The key feature of the proposed fluidic mixer is to generate air-bubbles within bulk solutions in a controllable manner. The size and number of the bubbles in the continuous flow are set by the relative flow rate between liquid phase and gas phase. Basically, the movement of bubbles in a straight PDMS-based fluidic channel depends on the number density of the bubbles, their sizes, their shapes, and their positions in the channel, which vary greatly among neighboring bubbles. The constant movements of bubbles with respect to one another create ever-changing liquid volume redistribution, and thus cause turbulent flow, favoring fast mixing. Suppose the simplest case is to solve the Navier-Stockes equation for lateral velocity *v_y_* in the flow (perpendicular to the flow direction) with the boundary Ω between the continuous flow phase and one individual bubble.

(1) $$\frac{\partial v_{y}}{\partial t}+\;v_{x}\frac{\partial v_{y}}{\partial x}\;+\;v_{y}\frac{\partial v_{y}}{\partial y}\;=\;-\frac{1}{\rho }\frac{\partial p}{\partial y}+\;v\;\left(\frac{\partial^{2}v_{y}}{\partial x^{2}}\;+\;\frac{\partial^{2}v_{y}}{\partial y^{2}}\right)$$

With the coupling of the boundary constriction in the normal and tangential components ${\vec{v}\;|}_{\Omega }=0,\;\vec{n}\cdot {\overset{=}{\tau }\;|}_{\Omega }=\kappa \gamma \vec{n}\;$, plus the incompressible assumption $\frac{\partial v_{x}}{x}+\frac{\partial v_{y}}{y}=0$, it is possible to solve these set of equations numerically based on computational algorithm for the similar situation of bubble rising in stationary liquid [30]. From the experimental point of view, obviously, the lateral convective velocity is enhanced by the introducing of bubbles, compared with that only by the thermal diffusion. The presence of groups of bubbles in the fluid allows for chaotic flow with the benefit for mixing. The bubbles change position, shape, and size, and therefore cause bubble swarming. To further enhance mixing, here we experimentally include the constrictions of various sizes into the design of the channel geometry, which makes it difficult to solve the analytics, even numerically, for the velocity field.

For instance, the interaction between bubbles and walls is tuned by the complex friction, and coupled deformation among bubbles by convergent-divergent constriction. Particularly, the deformation controlled by the competition of surface tension and velocity gradient makes it complex to find the detailed boundary, which involves solving the governing equation. However, from the competition among the viscous forces, inertia forces, and capillary forces in the bubbly flow, we can demonstrate how they determine the mixing in the channels.

### 3.2. The Fluidic Mixer

Based on above-mentioned conception, we fabricate a fluidic chip using 3D printing and the soft lithography techniques, which consists of only one poly-dimethylsioxane (PDMS) layer. The key elements of the fluidic mixer are the varying constriction dimension and a Y-shaped conjunction, where air bubbles are frequently generated (see Figure 1). The Y-channels are 0.5 mm deep and 1 mm wide with two branches connected to the solutes and air, respectively. With a constant input flow rate using a syringe pump, the air pressure determines the size of air-bubbles (Supplementary Section S1). As the generation of droplets can be disturbed by numerous factors (e.g., tube misplacement and deformation of PDMS channels when being pressurized), the bubble size varies randomly even at constant air-pressure. To create physical obstacles, the neck constriction is made 2 mm (wide) by 1 mm (deep) for the wide channels, and 0.5 mm by 1 mm for narrow parts.

### 3.3. Recirculating Flow in the Moving Droplet

**Figure 2:**
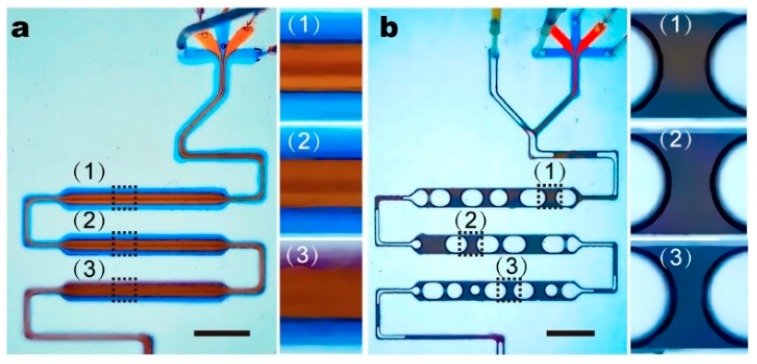
Comparison of mixing efficiency using food dyes between fluidic mixer with (**a**) laminar flow and (**b**) air-bubbles as stirrer. It is demonstrated that with air bubbles, food dyes of different colors can be quickly mixed. Scale bars denote 5 mm in all figures.

**Figure 3:**
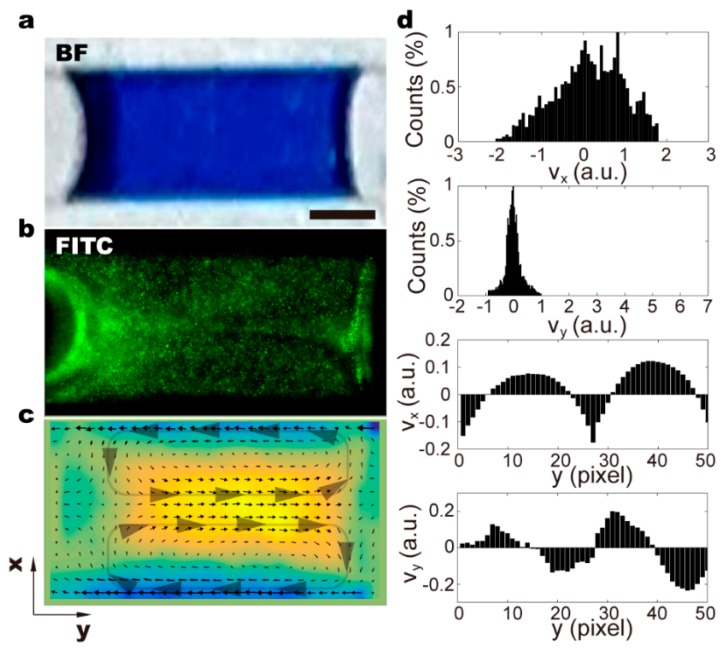
(**a**–**c**) 3D flow generated within the droplet in straight channels of the chip. It is demonstrated that circulating flow occurs within half of the droplet, and the mass transfer in the transversal direction still relies on molecular diffusion. (**d**) Distribution of flow rate within the moving droplet. The flow velocity is normalized to the driving flow rate to illustrate the mixing efficiency. We observe that flow distribution is symmetrical within a droplet, showing opposite flow profiles. The scale bar denotes 200 μm.

It is demonstrated that with no air input, solutes of the five inputs remain separated near the end of the fluidic chip (Figure 2a). The gradual blurry in the boundary from positions (1) to (3) can only be attributed to thermal dynamic diffusion and minor turbulent flow at the corner section. At high input air pressure, laminar streams of five solutions were frequently separated by air input and form individual droplets (Figure 2b). The mixing efficiency is satisfactory. Different coloring at the top and bottom parts of the droplet is still visible at position (1), which is ≈1 cm away from the Y-junction and position (2) (≈3 cm to the Y-junction). Apparently, it requires a certain distance for the droplet to move, which ensures thorough mixing. The flow profile is traced using fluorescent particles of ≈1 μm in diameter. We observe that the droplets moving in the straight parts of the fluidic channels generate a steady recirculating flow (Figure 3a–c) [31]. Even though the velocity perpendicular to fluidic channel can be as high as two times the driving flow rate, the direction distribution is symmetric, suggesting circulating flow within only half of the droplet (Figure 3d and Video S1). The flow rate along the channel direction is mostly slower than the driving flow rate. As reported before, the 3D flow allows mixing within half of the droplet, and thus the mass transfer in the transversal direction still relies on molecular diffusion and takes time [27,31]. The changed dimension of constrictions along the fluidic channel induces chaotic advection in individual droplets by dynamically changing the shape of the droplet and introducing asymmetry into the recirculating flow system [27]. As the convection flow rate is determined by droplet moving speed, the mixing efficiency depends strongly on the driving flow rate and channel geometry.

### 3.4. Small Air-Bubbles as Stirrers

**Figure 4:**
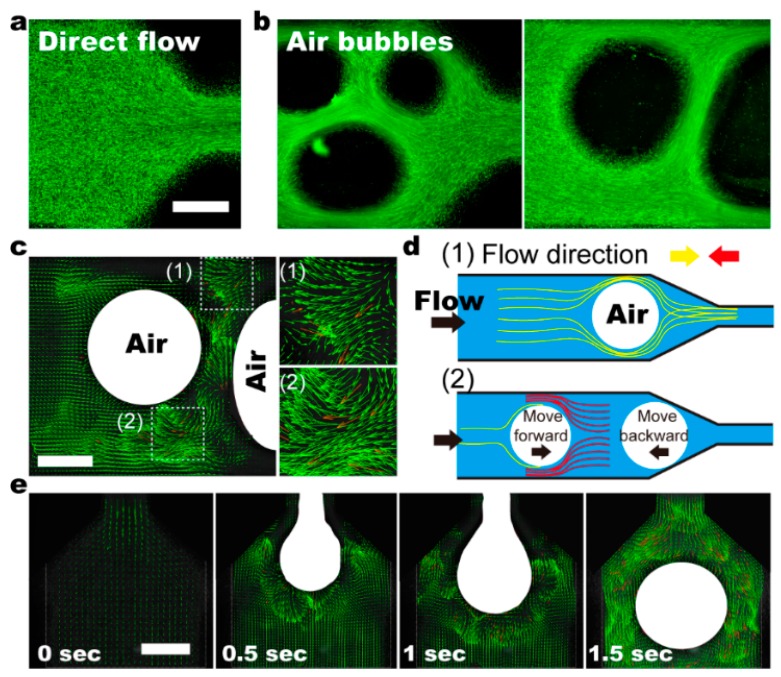
(**a**,**b**) Flow profile within the fluidic channels using air-bubbles (dark-circles) as the stirrer. Fluorescent particles of ≈1 mm in diameter are used as a flow tracer. (**c**) The flow profile around two air bubbles suggests turbulent flow and mass fluid transportation. (**d**) Schematic shows the mechanism of air bubbles working as the stirrer. (1) In the left panel, the existence of air bubble changes the channel cross-sectional area, and thus induces strong shear around the bubble. (2) The movement of air bubbles can be stopped by changed constriction geometry along the way. Meanwhile, the motion of the following bubbles remains, which changes the relative positions between neighboring bubbles. Consequently, liquid volume redistribution causes turbulent flow, and thus, fast mixing. (**e**) Time series showing the turbulent flow generated when an air bubble moves out from a narrow channel. Scale bars denote 500 μm in all figures.

Small bubbles with diameters ranging from 0.1 to 0.3 mm are generated in the fluidic channels at low air pressure (0.05 to 0.1 psi) (Figure 4). For each experiment, the input air-pressure is maintained at a constant value. As mentioned before, the variances in droplet size can be caused by minor disturbances from the environment. As the height of the free droplet is considerably higher than the fluidic channel (i.e., 1 mm), the droplets show an elliptical shape when being confined in the channel, resulting in large contact surface between bubbles and the channel walls. Like stranding pillars, these small bubbles firstly create physical obstacles for the laminar flow (Figure 4a), directing stream lines in the cross-sectional direction through constrictions (Figure 4b). The perturbed laminar flow thus enhances mixing capacities by generating stronger shear and more frequent chaotic advection (Figure 4c).

Notably, these small bubbles move with the bulk fluid (Video S2). When passing through constrictions, strong shear generated at the sides further facilitates mixing, preventing the fluidic channels from being fully blocked. Additionally, bubbles regain their round-shaped conformation when being “pushed” out (Figure 4e). The sudden changes in bubble shape and confinement space create chaotic advection, and facilitate mixing.

When groups of bubbles transporting in fluidic channels, there are three forces at play: viscous forces, inertia forces, and capillary forces. The ratio of any two gives three dimensionless numbers: Reynolds number *R_e_*, comparing inertia to viscos forces; capillary number *C_a_*, comparing viscous to capillary forces; and Weber number *W_e_*, comparing inertia to capillary forces. The detailed expressions are shown below,

(2) Re=ρUL/μ

(3) Ca=μU/γ

(4) $$We=\rho U^{2}L/\gamma$$

The typical bubble size is around one millimeter in diameter, which gives the characteristic length *L* ≈ 1 mm. The typical velocity of bubbles is around millimeter per second, which gives the characteristic velocity *U* ≈ 1 mm/s. The values of density, viscosity, and surface tension are taken from an aqueous solution, leading to *R_e_* ≈ 1, *C_a_* ≈ 10^−4^–10^−5^, and *W_e_* ≈ 10^−4^–10^−5^.

The bubbles are deformed by the competition between viscous driving forces enacted by surrounding fluids and resistant surface tension. The transverse velocities are caused by the inclusions, deformations, and slip boundaries of bubbles. A group of bubbles in the fluid lead to chaotic flow, benefiting mixing. The varying of bubble positions, shapes, and sizes can therefore cause complex bubble swarming. Note that the interaction between bubbles and channel walls in direct contact, due to the friction, slows down or even trap bubbles, leading to the deformation of streamlines. Besides, the diverging part of the channel creating a compressive flow results in an instability of particles’ positions downstream.

**Figure 5:**
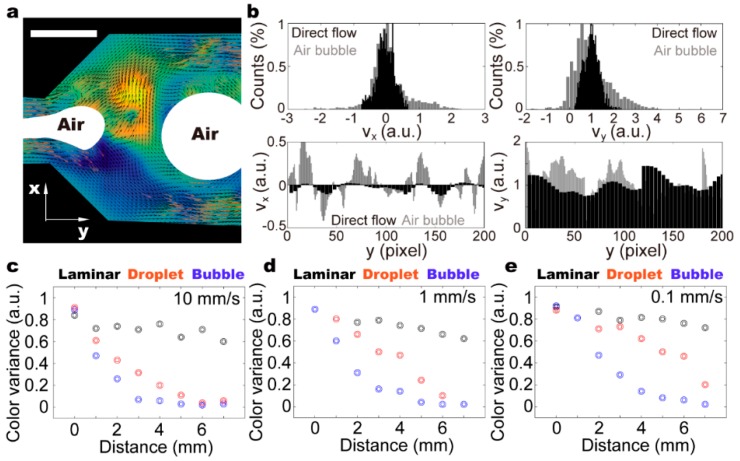
(**a**) Turbulent flow generated with the presence of air bubbles on-chip. (**b**) Flow distribution within the channels demonstrates that air bubbles as stirrers substantially enhance the flow rate in cross-sectional direction of the channel, which allows for fast mixing. The flow velocity is normalized to the driving flow rate to illustrate the mixing efficiency. (**c**–**e**) Color variances are measured in the cross-section at different positions along the fluidic channel (i.e., distance from the Y-junction). To obtain the color variances, light intensities of selected area along the fluidic channel are measured in the red channel of the RGB picture, and calculated to get the standard deviation (Figure S3). Three different driving flow rates (i.e., 10, 1, and 0.1 mm/s) are analyzed. The scale bar denotes 500 μm.

Consequently, the transportation speeds of air-bubbles in a fluidic channel are different even when they are similar in size. The altered relative positions of air-bubbles cause liquid volume redistribution, and thus, chaotic advection (Figure 5a and Video S2). It is demonstrated that within a selected area, the velocity perpendicular to fluidic channel can be as large as two times the driving flow rate, which is on par with the recirculating flow in the moving droplet (Figure 5b). The flow distribution, however, is highly asymmetrical, suggesting directional flow across the whole channel. Intriguingly, the velocity along the channels can be four times larger than the driving flow rate, which is considerably higher than both the laminar flow and droplet mixing. Therefore, we conclude that the flow distribution with small bubbles as stirrers is ever-changing and mostly random. Using food dyes of different colors, we assess the mixing capacities of laminar flow, droplet, and bubble (Figure 5c–e). It is demonstrated that in contrast to the laminar flow, both droplets and bubbles can ensure thorough mixing within a fluidic chip. The mixing efficiency of a droplet depends significantly on the driving flow rate, and the bubbles as stirrers in a bulk solution quickly generate fully mixed solutions even at a low driving flow rate (i.e., 0.1 mm/s). Our results suggest that bubbles as an active stirrer (i.e., self-orientation) can generate strong chaotic advection, even within a straight channel, which is a key attractive feature of the proposed fluidic mixer.

## 4. Conclusions

In this study, we chose not to use any external field to achieve active mixing, which allows the system to be easily fabricated and operated. Instead, chaotic advection is induced using air-bubbles as stirrers and varying channel geometry as physical obstacles. For biological and biomedical applications, in which air-bubbles can cause significant effects on the on-chip culture environment, a degassing unit can be integrated to remove the unwanted gas-liquid interface (Figure S2 and Video S3). Our proposed fluidic mixer possesses the characteristics of both active and passive mixers, allowing effective solute mixing even at a relatively low input flow rate. We believe that it will be useful for both chemical analysis and synthesis, and for studies of complex reaction networks.

## Supplementary Materials

The following are available online at https://www.mdpi.com/2072-666X/11/2/195/s1. Figure S1: Fabrication process of the fluidic mixer. The template is fabricated using 3D printing. The mixed PDMS solution (base and curing agent, 10:1) is transferred into the template, which is followed by incubated at 45 °C for 24 h. The cured PDMS is then sealed with glass slides through plasma etching. Figure S2: (**a**) Optical image and (**b**) schematic showing that the air-bubbles can be effectively removed by integrating a degassing unit into the fluidic chip. (b) By collecting the mixed solution into an empty chamber, the gas and liquid can be quickly separated due to gravity. Figure S3: (**a**,**c**) Original optical images and (**b**,**d**) mono-color (red) images of the fluid channel. (**e**) Histogram of the selected area marked in Figure (**b**). (**f**) Histogram of the selected area marked in Figure (**d**). It is demonstrated that with air-bubbles as stirrers, the intensity distribution can become significantly narrower. Video S1: Movement of a droplet within a straight fluidic channel. Video S2: Chaotic advection caused by the movement of small droplets within the bulk solution. Video S3: Operation of a fluidic mixer chip integrated with a degassing unit. It is demonstrated that the air-bubbles are trapped in the large reservoir, and the “clear” mixed solution is collected elsewhere.
