# Supplementary material for: Air-Bubble Induced Mixing: A Fluidic Mixer Chip
Source: Micromachines (Basel). 2020 Feb 14;11(2):195. doi: 10.3390/mi11020195 (PMC7074661; doi:10.3390/mi11020195)
Supplement: Supplementary file 1 [file micromachines-11-00195-s001.zip › micromachines-699640-supplementary-for final/micromachines-699640-supplementary-for final.docx]

Supplementary Materials

Air-Bubble Induced Mixing: A Fluidic Mixer Chip

**Xiaoyu Jia, Bingchen Che, Guangyin Jing and Ce Zhang**

1. Correlation between Air-Bubble Size and the Controlling Parameters

To make the problem simpler, we try to make a discussion on the scaling analysis for the controlling parameters to evaluate air-bubble size; i.e., the correlation between bubble size and the driving flow rate, channel dimensions, and the nature of the mixing solution. As indicated in
Figure R1, the continuous flow produces air-bubbles from the air inlet at the adjacent of the Y junction. The volume of the air-bubble produced is determined by the balance between the pressure difference across the bubble diameter and the Laplace pressure (shown in the Figure S4). Within the channel (width *w* and height *h*), this driving pressure reads $\Delta p \sim R_{H}Q_{b}= {12\mu\cdot2RQ_{b}}/{h^{3}w}$, where $R_{H}, \mu, Q_{b}$are hydrodynamic resistance, effective viscosity, and flow rate of the mixing solution, respectively. The Laplace pressure $p$ is determined by the surface tension $\gamma$ of the mixing solution and the size $R$ of the air bubble, i.e., $p\sim2\gamma/R$, which is maintained by the pressure controller. With the surface tension $\gamma\sim0.05 N/m$ and R ≈ 0.1 mm in our cases, the Laplace pressure is estimated, which is on the same order of pressure used in our experiments. Therefore, the bubble size has the relation of $R \sim(\left( \gamma/\mu\right)\cdot\left( {{wh}^{3}}/{{12Q}_{b}} \right))^{1/2}$.


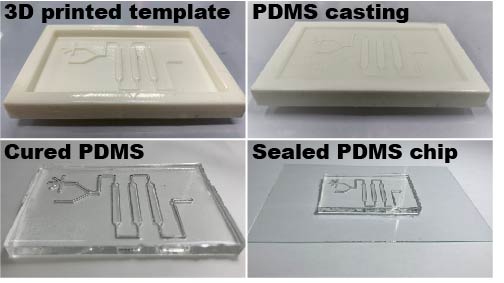


**Figure S1.** Fabrication process of the fluidic mixer. The template is fabricated using 3D printing. The mixed PDMS solution (base and curing agent, 10:1) is transferred into the template, which is followed by incubated at 45 °C for 24 h. The cured PDMS is then sealed with glass slides through plasma etching.


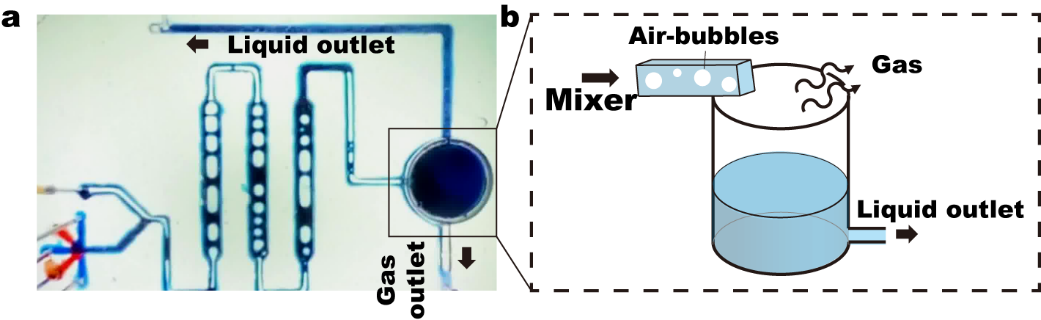


**Figure S2.** (**a**) Optical image and (**b**) schematic showing that the air-bubbles can be effectively removed by integrating a degassing unit into the fluidic chip. (**b**) By collecting the mixed solution into an empty chamber, the gas and liquid can be quickly separated due to gravity.


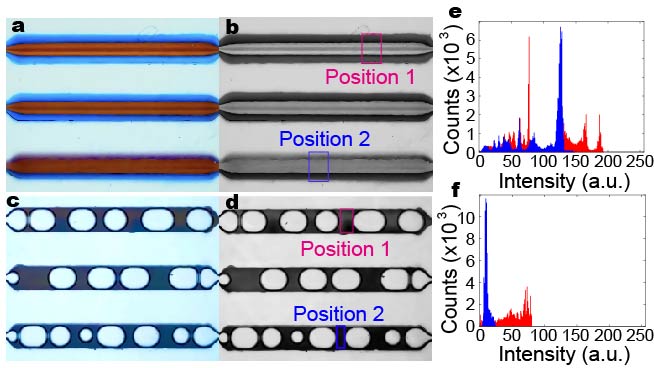


**Figure S3.** (**a**,**c**) RGB and (**b**,**d**) mono-color (red) images of the fluid channel. (**e**) Histogram of the selected area marked in Figure (**b**). (**f**) Histogram of the selected area marked in
Figure (**d**). It is demonstrated that with air-bubbles as stirrers, the intensity distribution can become significantly narrower.


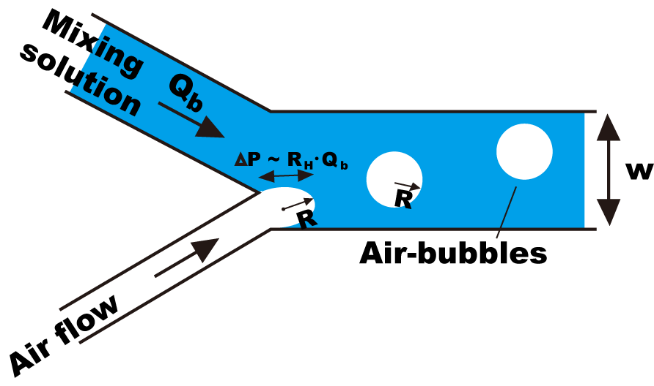


**Figure S4.** Schematic showing the production of air-bubbles within the bulk solution using a Y-shaped junction. The size of the air-bubbles *R* can be controlled by adjusting the solution flow rate $\boldsymbol{Q}_{\boldsymbol{b}}$ and air input pressure.

Video S1: Movement of a droplet within a straight fluidic channel.

It is demonstrated that recirculating flow can be generated, allowing solute exchange only in half of the droplet

Video S2: Chaotic advection caused by the movement of small droplets within the bulk solution.

We observe that the movement of small droplet with respect to one another causes liquid volume redistribution, and thus the chaotic advection. Strong shear can also be generated through bubble deformation.

Video S3: Operation of a fluidic mixer chip integrated with a degassing unit.

It is demonstrated that the air-bubbles are trapped in the large reservoir, and the “clear” mixed solution is collected elsewhere.

| 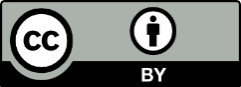 | © 2020 by the authors. Submitted for possible open access publication under the terms and conditions of the Creative Commons Attribution (CC BY) license (http://creativecommons.org/licenses/by/4.0/). |
| --- | --- |
